# Early visual motion experience shapes the gap junction connections among direction selective ganglion cells

**DOI:** 10.1371/journal.pbio.3000692

**Published:** 2020-03-25

**Authors:** Li Zhang, Qiwen Wu, Yifeng Zhang

**Affiliations:** 1 Institute of Neuroscience, Chinese Academy of Sciences, Shanghai, China; 2 University of Chinese Academy of Sciences, Beijing, China; Yale University, UNITED STATES

## Abstract

Gap junction connections between neurons play critical roles in the development of the nervous system. However, studies on the sensory experience–driven plasticity during the critical period rarely examine the involvement of gap junction connections. ON-OFF direction selective ganglion cells (ooDSGCs) in the mouse retina that prefer upward motion are connected by gap junctions throughout development. Here, we show that after exposing the mice to a visual environment dominated by upward motion from eye-opening to puberty, ooDSGCs that respond preferentially to upward motion show enhanced spike synchronization, while downward motion training has the opposite effect. The effect is long-term, persisting at least three months after the training. Correlated activity during training is tightly linked to this effect: Cells trained by stimuli that promote higher levels of activity correlation show stronger gap junction connection after the training, while stimuli that produce very low activity correlation leave the cells with much weaker gap junction connections afterwards. Direct investigation of the gap junction connections among upward motion–preferring ooDSGCs show that both the percentage of electrically coupled ooDSGCs and the strength of the coupling are affected by visual motion training. Our results demonstrate that in the retina, one of the peripheral sensory systems, gap junction connections can be shaped by experience during development.

## Abstract 2

Gap junction connections between upward motion–preferring direction selective ganglion cells can be shaped by early visual experience; upward motion training leads to enhanced connectivity, while downward motion greatly suppresses the connection, suggesting a form of activity-dependent plasticity.

## Introduction

Gap junction connections, also called electrical synapses, between neurons play critical roles in the development of the nervous system [1–4]. Gap junction connections are widely distributed in the mammalian retina [5–9]. They also show an extensive range of plasticity that helps in the processing of visual information, such as modulating circuit function based on light levels [6,10–13] and mediating correlated firing in a stimulus-dependent manner [14,15]. However, whether some types of plasticity exist during the critical period to alter the neural circuits based on sensory experience remains largely unexplored.

Direction selective ganglion cells (DSGCs) in the retina are important in the processing of motion information. The development of the ON-OFF direction selective ganglion cells (ooDSGCs) and the role of visual experience in it have been extensively studied [16–20]. The direction selectivity (DS) of ooDSGCs in the mouse retina is already established before eye-opening (between postnatal days [P]12 and P14), and is not dependent on visual experience [17,21]. However, the proper realignment of the preferred directions is delayed in the absence of visual experience [20], suggesting some plasticity in the direction selective circuits still exists after eye-opening. The ooDSGCs can be divided into four subtypes [17,22], each preferring a different direction of motion: ventral, dorsal, temporal, and nasal direction on the retina (V-DSGC, D-DSGC, T-DSGC and N-DSGC, respectively). Tracer coupling experiments indicate that all four subtypes have gap junction connections with the same subtype as well as between V- and T-DSGCs before eye-opening, but N- and D-DSGCs quickly decouple by P12, and T-DSGCs decouple by P15, whereas V-DSGCs remain coupled until adult age, possibly throughout the adult life [19]. Why gap junction connections are preserved specifically in V-DSGCs is not yet clear [23]. Nevertheless, this provides us an opportunity to study if electrical coupling among V-DSGCs can be altered by a biased visual experience during the critical period.

We trained the mice with a visual environment that was dominated by a single direction of motion from eye-opening to the end of puberty, then tested whether such training had an impact on the gap junction connections between ooDSGCs. Our results suggest that the gap junction connections in ooDSGCs can be permanently enhanced by early exposure to motion stimuli that induce high levels of correlated activity or suppressed by training with stimuli that induce low or no correlated activity.

## Results

### Functional electrical coupling among V-DSGCs during development

**Fig 1:**
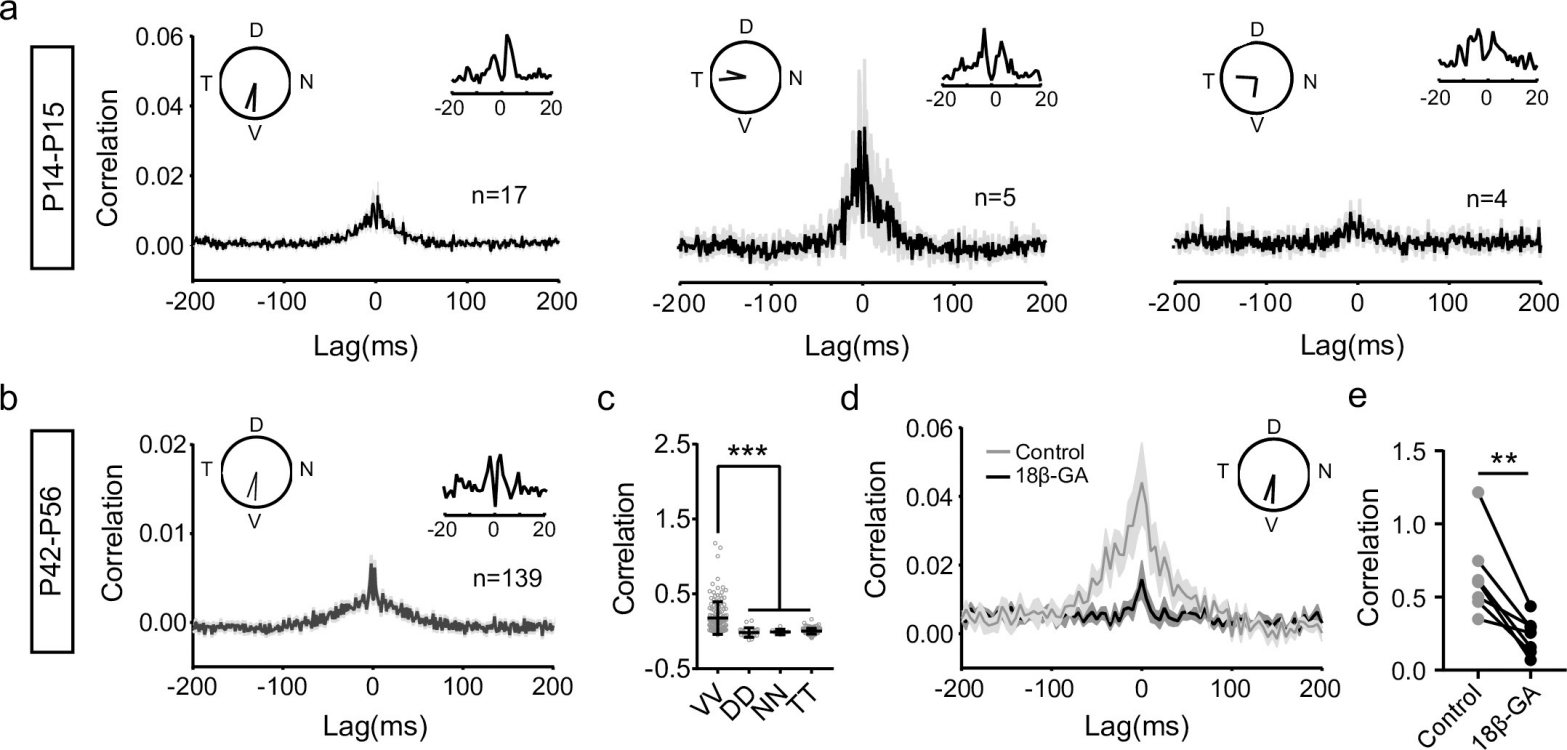
Electrical coupling among ooDSGCs during development. (a) Spike time cross-correlograms for ooDSGC pairs in the juvenile mice (P14–P15). Solid black line, average cross-correlogram across all recorded pairs of the specified combination. Shaded area, SEM. For each panel, the inset on the left indicates the ooDSGCs pair; inset on the right is a close-up view of the cross-correlogram with 1-ms bin size. The total numbers of recorded pairs are also indicated on the graph. (b) The same as (a), for V-DSGC pairs in adult mice (P42–P56). (c) Comparison of total spike time correlation among V-V, D-D, N-N, and T-T DSGC pairs in adult mice. Only V-V pairs show significant correlated activity. V-V, *n* = 139; D-D, *n* = 22; N-N, *n* = 8; T-T, *n* = 172. ***p < 0.001. Error bars: SD. (d,e) Gap junction blocker 18β-GA largely blocks the correlated activity among V-DSGCs. (d) Comparison of the average cross-correlograms. Gray, control; black, 18β-GA. *n* = 7 pairs. Bin size, 5 ms. (e) Paired comparison of the correlation using the same data as in (d). Correlation is calculated as the sum of cross-correlation from −100 ms to 100 ms with 1-ms time bin. For every pair tested, a reduction in correlated activity is induced with 18β-GA. **p < 0.01, paired *t* test. Data for this figure are in S1 Data. ooDSGC, ON-OFF direction selective ganglion cell; T, temporal-preferring direction selective ganglion cell; V, ventral-preferring direction selective ganglion cell; 18β-GA, 18β-glycyrrhizic acid.

Tracer coupling experiments have revealed the presence of gap junctions in ooDSGCs around eye-opening [19]. We first confirmed that these were indeed functional electrical couplings. Gap junction connections are known to be related to correlated activity between neurons [2,24]. We thus recorded ooDSGCs in C57 mice at different ages using multielectrode arrays (MEAs) and analyzed spike time correlation for each ooDSGC pair. There were much higher incidences of V/V and T/T pairs compared to D/D and N/N pairs. This was due to the about 2-fold higher proportions of V- and T-DSGCs than the N- and D-DSGCs in our recording. Similar results have been reported elsewhere and were suggested to be due to the sampling bias of the MEA method [17,25]. Such a sampling bias should not affect our spike time correlation analysis. In juvenile mice a couple of days post eye-opening (P14–P15), some V-DSGCs pairs showed significant correlation at a timescale of around ±30–50 ms (Fig 1A left). In addition, for many pairs, two sharp peaks at around ±3–5 ms existed, evidence of direct electrical coupling between V-DSGCs (Fig 1A left inset). The same form of synchronized activity could be observed for T-DSGC pairs (Fig 1A middle) and for T/V-DSGC pairs (between a T-DSGC and a V-DSGC, Fig 1A right). For pairs that involve the other two ooDSGC subtypes, no significant correlation of spike times at any timescale was detected. In more developed retinas (P42–P56), only V-DSGCs showed significant correlation of activity (Fig 1B and 1C and S1 Fig). The observed pattern of spike time correlation is gap junction dependent, as it can be blocked by a gap junction blocker, 18β-glycyrrhizic acid (18β-GA; Fig 1D and 1E). These results show that after eye-opening, when visual experience is available to exert an influence on the development of the visual system, V-DSGCs are electrically coupled.

### Training with motion impacts synchronized activity in V-DSGCs

**Fig 2:**
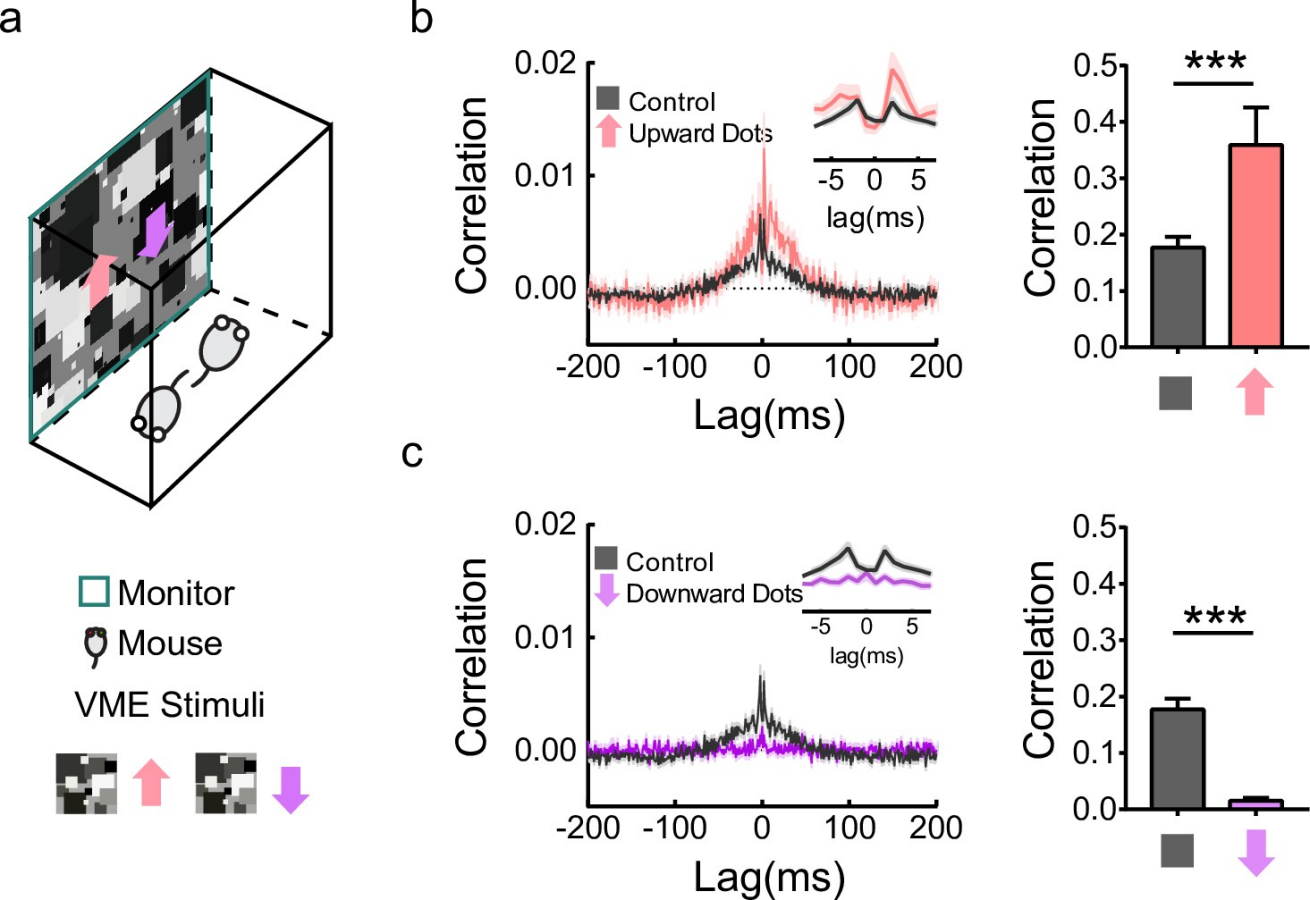
VME affected synchronized activity in V-DSGCs. (a) Diagram of the VME training (top) and the color codes for VME stimuli (bottom). For each stimulus, left, spatial pattern; right, color code with direction of motion. (b) Average cross-correlogram of V-DSGC pairs during travelling gratings stimulus (left) and quantification of correlation strength (right). Control, gray, *n* = 139 pairs of V-DSGCs; upward VME, pink, *n* = 51. Shaded areas, ±SEM. Insets are close-up views with 1-ms bin size. For correlation strength comparison, the sum of the spike time cross-correlation (left) from −100 ms to +100 ms at a bin size of 1 ms are used. ***p < 0.001, unpaired *t* test. Error bars, SEM. (c) The same as (b) but to compare the control group with the downward VME group. Control, gray, *n* = 139; downward VME, purple, *n* = 130. For correlation strength comparison, ***p < 0.001, unpaired *t* test. Error bars, SEM. Data for this figure are in S2 Data. DSGC, direction selective ganglion cell; VME, visual motion experience.

Visual motion experience (VME) was provided to the mice for 12 hours each day during an important period for visual system development [26] from P10 to P35. The motion experience consisted of dots of various sizes, contrasts, and speeds, moving either in upward direction, to best stimulate the V-DSGCs, or in the opposite downward direction (Fig 2A). Control mice were reared in home cages with normal visual experience. All tests were performed after P35. If the gap junction connections among V-DSGCs are altered by the early VME, we expect to observe a change in the level of synchronized firing.

We first examined if total correlated activity among V-DSGCs were different between mice trained with VME (VME groups) in a single direction and mice with no training (control group). Average spike time correlation for the V-DSGC pairs was significantly enhanced in the upward VME group (Fig 2B) but suppressed in the downward VME group (Fig 2C). In fact, almost no spike synchrony was observed in the downward VME-trained V-DSGCs. Note that the lens reverses the image, and thus upward direction, when projected onto the retina, is the preferred direction for V-DSGCs and strongly activates them, while downward motion is their null direction. Hence, VME training using a motion stimulus that best activates the V-DSGCs induced an increase in spike time correlation, possibly due to enhanced gap junction connectivity, while a training stimulus in the opposite direction achieved the opposite effect: greatly diminished spike time correlation. If gap junction coupling is behind such changes, then spike time correlations of spontaneous activity should also change. Cross-correlograms of V-DSGCs in the absence of patterned visual stimuli indeed show similar change to what we observed using a motion stimulus (S2 Fig), substantiating that the effects were not due to the temporal structure of the specific stimulus presented to the retina but likely a general change in gap junction connections among V-DSGCs.

### The level of correlated activity during training underlies the changes

**Fig 3:**
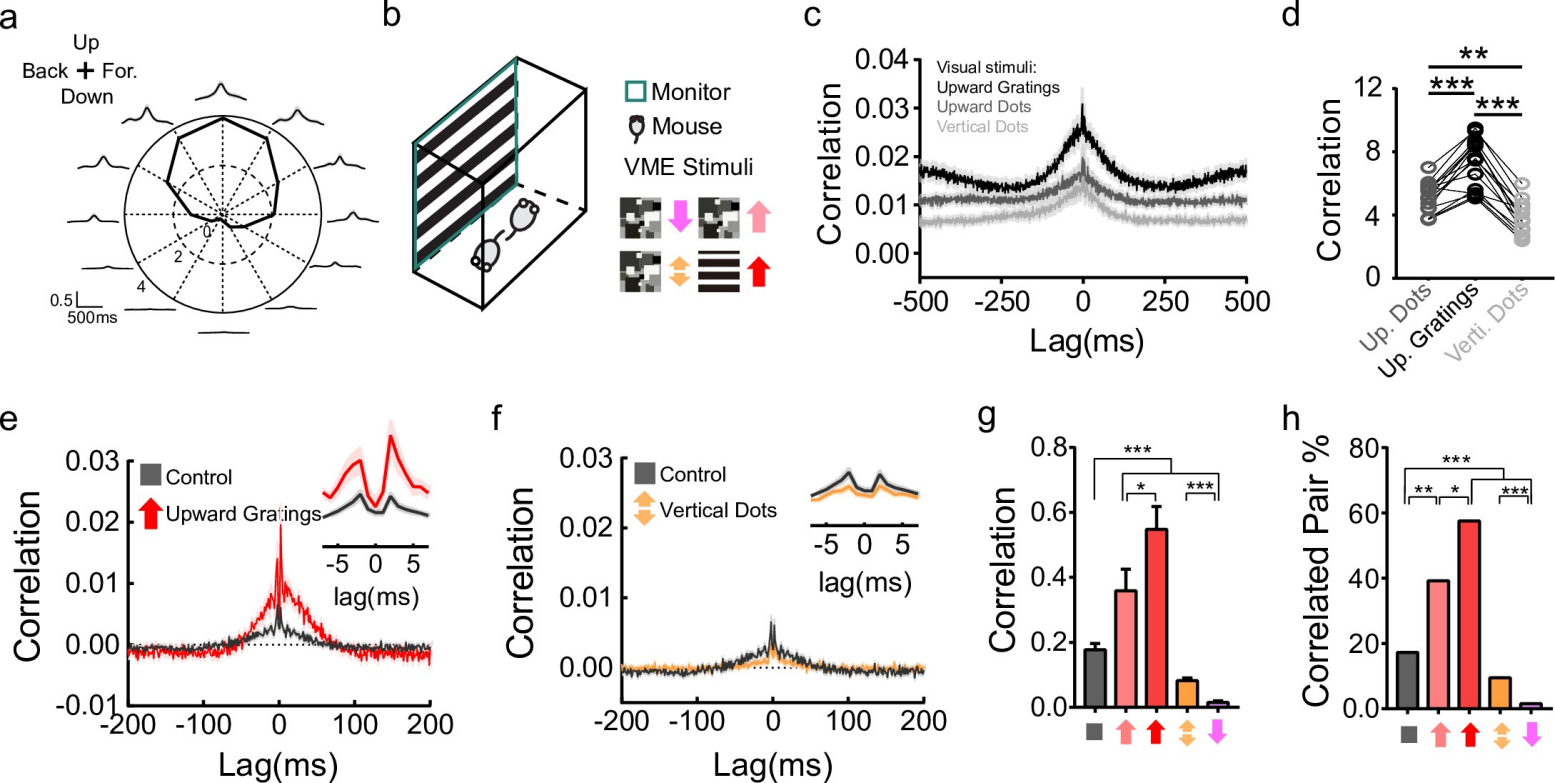
Correlated activity underlies VME-induced changes in ooDSGCs. (a) Polar plot and cross-correlograms showing levels of correlated activity (averaged across V-DSGC pairs) induced by different directions of motion stimuli. Upward motion induces the highest spike time correlation; downward motion induces almost no spike time correlation among V-DSGCs. Stimulus: the same stimulus as used for upward and downward dots VME, except also with motion in 10 other directions. Cross-correlation calculation was done with a 25-ms time bin. Stimulus effect is not removed. (b) Illustration of the VME stimuli that induce different levels of correlated activity. For each stimulus, left, spatial pattern; right, color code with direction of motion. (c) Spike time cross-correlograms under three different visual stimuli: upward gratings (red), upward dots (pink), and vertical dots (orange). Solid lines, average cross-correlograms; shade areas, ±SEM. Only cross-correlograms for V-DSGC pairs are included. Note the cross-correlograms shown here intentionally do not remove the effect of the stimuli, to best represent the amount of difference in total correlated activity elicited by these stimuli. (d) Comparison of total correlated activity evoked by three visual stimuli shown in (c). Correlation is computed as described in 2d. *n* = 16 pairs; ***p < 0.001, **p < 0.01, paired *t* test. (e,f) Average cross-correlogram of V-DSGC pairs after VME training with upward gratings (e) and vertical dots (f). Cross-correlograms of all V-DSGC pairs are averaged. Responses used to calculate the cross-correlograms were recorded during travelling gratings. Control, gray, *n* = 139; upward VME, red, *n* = 33; vertical VME, orange, *n* = 253; shaded areas, ±SEM. Insets are close-up views with 1-ms bin size. (g) Quantification of correlation strength from cross-correlograms in 2b, 3e, and 3f. Correlation is computed as described in 2d. *p < 0.05, ***p < 0.001, unpaired *t* test. Error bars, SEM. (h) Proportion of V-DSGC pairs with measurable gap junction connections. The proportions of correlated pairs in all V-DSGC pairs show significant difference, with upward VMEs higher than control and vertical VME lower than control (chi-squared test, ***p < 0.001; **p < 0.01). In addition, the upward gratings VME is significantly higher than upward dots VME (chi-squared test, *p < 0.05). Data for this figure are in S3 Data. ooDSGC, ON-OFF direction selective ganglion cell; VME, visual motion experience.

We next asked what aspect of the VME training is involved in inducing the changes in correlated activity after the training. Because upward motion is the preferred direction and downward motion is the null direction for V-DSGCs, one obvious possibility is the activity level of V-DSGCs during VME training. Alternatively, V-DSGCs are electrically coupled to each other, so upward and downward motion may evoke different levels of activity correlation. We directly tested this using the same random dots motion used for the VME trainings and recorded the response of untrained V-DSGCs (Fig 2A). The spike time cross-correlation between V-DSGC pairs under this stimulus was computed. In this case, we did not remove the effect of the stimulus by subtracting cross-correlation from shuffled trials (also known as the shift predictor). The idea was to compare the total amount of synchronized activity, not gap junction connectivity. We surmised that V-DSGCs that tended to fire more synchronously during development might have gap junction connections between them potentiated, and vice versa. Indeed, downward motion induced very low activity correlation in V-DSGCs, while upward motion induced the strongest activity correlation (Fig 3A). This was not simply due to the difference in firing rates. When we removed the effect of firing rates from the cross-correlation by normalizing it to the product of the numbers of spikes [27], the same difference in activity correlation was preserved (S3 Fig). Thus, different directions of motion stimuli trigger different levels of correlation in V-DSGCs. Consequently, upward VME training leads to highly correlated firings in V-DSGCs during post eye-opening development, while downward VME training induces a very low level of activity correlation.

If correlated activity through gap junctions during VME underlies the changes in activity correlation among V-DSGCs later, then VME stimuli that are similar to what we used before but elicit different levels of correlated activity should be able to produce different effects. We first tested a vertical moving dots stimulus (Fig 3B). The stimulus was a combination of the upward and downward dots stimuli, with every dot on screen having a 50% chance of moving either upward or downward. Upward motion strongly excites V-DSGCs, whereas downward motion only weakly excites or even inhibits V-DSGCs [28,29]. With such a VME stimulus, at any given moment, a random proportion of about 50% of V-DSGCs are strongly activated and the other 50% are not, thus reducing activity correlation in the V-DSGCs compared to those induced by upward dots. This was confirmed by measuring V-DSGCs’ actual spike time correlation during this stimulus (Fig 3C and 3D). After the vertical dots stimulus was used for VME training, significant decrease in synchronized firing is observed, although the change is not as dramatic as in the case of downward motion alone (Figs 2C, 3F and 3G).

A VME stimulus that induces higher correlated activity was also tested. This stimulus consists of square wave gratings that move in the upward direction (Fig 3B). Periodically, the spatial frequency, temporal frequency, and contrast of the gratings change in random combinations so that, over time, these parameters cover roughly the same range as the previously used moving dots stimuli. At any given moment, however, this stimulus is much more spatially and temporally coherent than the moving dots stimuli, thus inducing higher correlated activity (Fig 3C and 3D) but roughly the same mean firing rate in V-DSGCs (S1 Table, row 1). When it was used as the VME training stimulus, again we see a significant increase in activity correlation among V-DSGCs compared to control. Both upward gratings and upward dots VME induce stronger activity correlation on average, with a higher percentage of V-DSGC pairs showing correlation compared to control (Fig 3G and 3H). Furthermore, the changes in correlation and the number of correlated V-DSGC pairs are both significantly higher for upward gratings compared to upward dots, indicating upward gratings is a more effective stimulus than dots to induce changes in activity correlation, despite the fact that they elicit roughly the same level of activity in V-DSGCs.

**Fig 4:**
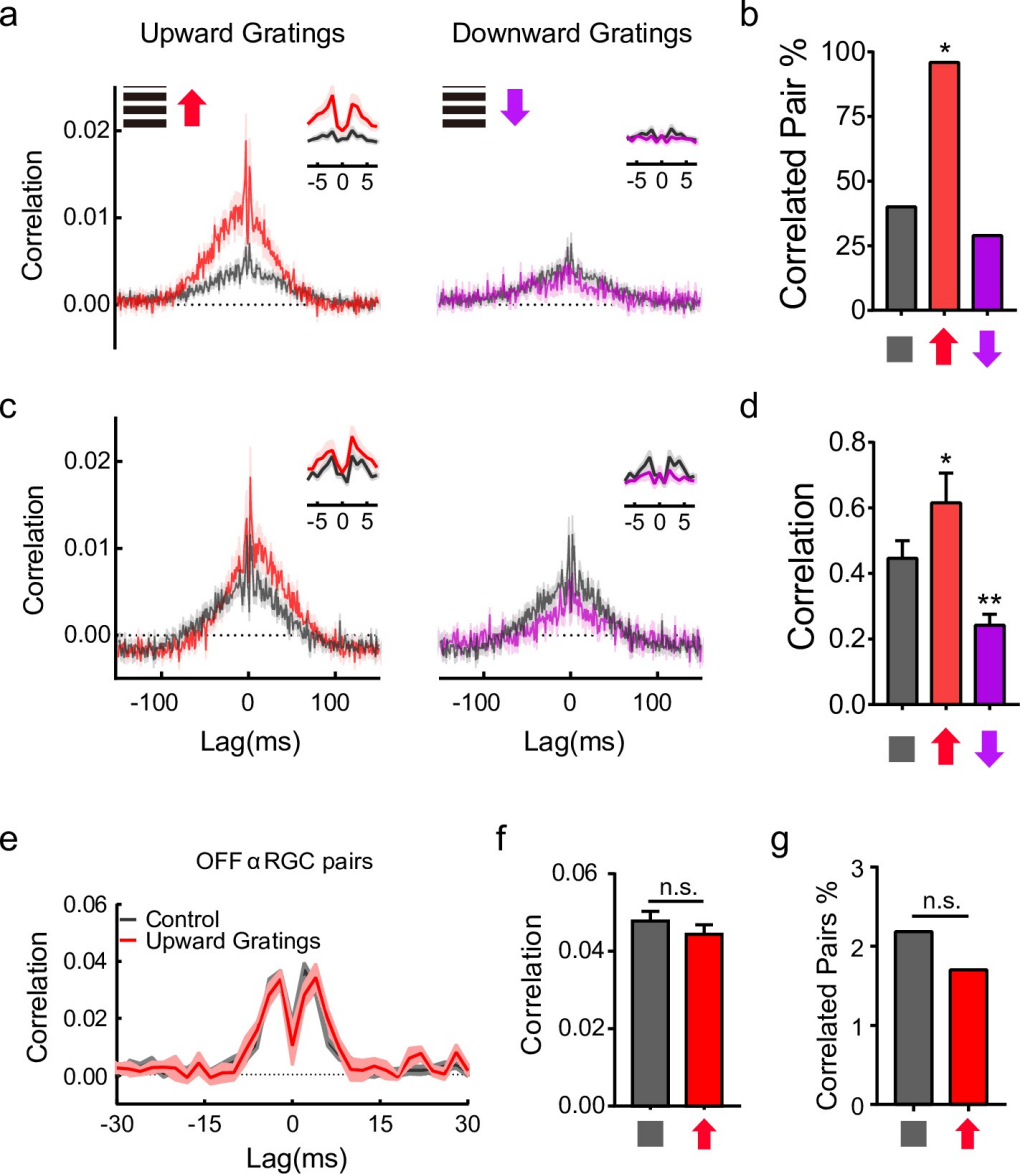
Proportion of correlated pairs and correlation strength are both affected by VME. (a) Average cross-correlogram of V-DSGC pairs at a close distance. Cross-correlograms of all V-DSGC pairs within 250 μm of each other are averaged. Control, gray, *n* = 75; upward VME, red, *n* = 24; downward VME, magenta, *n* = 38; shaded areas, ±SEM. Insets are close-up views with 1-ms bin size. (b) Proportion of V-DSGC pairs with measurable activity correlation in different groups shown in (a), with the same color codes. Control/upward VME/downward VME: *n* = 75/24/38 pairs. The proportion of correlated pairs show significant difference between the control group and the upward VME group. Chi-squared test, *p* = 0.01 for upward VME group, *p* = 0.48 for downward VME group. (c) The same as in (a), except that, of the V-DSGC pairs represented in (a), only those with significant spike time correlation are used for averaging. Control, gray, *n* = 30; upward VME, red, *n* = 23; downward VME, magenta, *n* = 16; Shaded areas, ±SEM. (d) Quantification of correlation strength from cross-correlograms in (c), computed as described in 2d. **p < 0.01, *p < 0.05, unpaired *t* test. Error bars, SEM. (e, f, g) Comparison of the gap junction connections between OFF αRGC pairs. (e) Average cross-correlograms of all correlated OFF αRGC pairs. (f) Quantification of total correlation strength based on (e). The sum of the spike time cross-correlation in (e) from −15 ms to +15 ms is used for comparison. No significant change in the strength of correlation can be detected, *p* = 0.52, unpaired *t* test. (g) Proportion of correlated OFFα RGC pairs show no difference between control and upward VME. Chi-squared test, *p* = 0.53. Control, gray, *n* = 45 pairs; upward gratings VME, red, *n* = 11 pairs. Shaded areas, ±SEM. Data for this figure are in S4 Data. αRGC, alpha retinal ganglion cell; V-DSGC, ventral-preferring direction selective ganglion cell; VME, visual motion experience.

The VME group using downward-moving gratings for training was also analyzed and found for the most part to be indistinguishable from the control (Fig 4A and 4B). A downward gratings stimulus generates stronger activity correlation than downward dots. Correspondingly, compared to downward dots VME, which led to almost complete abolishment of V-DSGC synchrony, downward gratings training left V-DSGC coupling mostly the same as control (Fig 4A and 4B). It is also worth noting that a vertical dots stimulus generates stronger activity in V-DSGCs than a downward gratings stimulus (S4 Fig), yet VME training with the former suppressed the spike time correlation strongly, while the latter left it unchanged. This further reinforces the notion that the level of activity during VME training is not directly linked to its effect on correlated firing.

Together, these results show that by manipulating the level of synchronized firing during VME training, one can influence synchronized firing among V-DSGCs after the training is terminated. Meanwhile, the absolute level of activity in V-DSGCs during training is likely not directly linked to this change in synchronized firing.

### Higher proportion of synchronized cells, stronger synchronization, and long term

We looked at the proportion of synchronized V-DSGC pairs more closely, focusing on VME groups trained by upward gratings, with downward gratings VME as control. If we counted all V-DSGCs pairs, regardless of their distance from each other, we found a nearly 3-fold increase in the percentage of coupled pairs in upward VME retinas compared to control, whereas downward VME induced no such change. The difference was perhaps more remarkable for pairs that were close to each other (Fig 4A): nearly all pairs with a maximum of 250 μm distance between somas in the upward gratings VME group had significant spike time correlation, while this number is only about 40% and 25% in the control and downward VME groups, respectively (Fig 4B).

Can this increased number of coupled V-DSGC pairs account for all the observed elevation in correlated activities among these cells, or is coupling strength also affected by VME? We identified close V-DSGC pairs (soma distance <250 μm) that have statistically significant amounts of correlated activity. When only these “correlated pairs” were counted, the average spike time correlation per V-DSGC pair for upward VME groups is slightly but significantly larger than control (Fig 4C and 4D), suggesting upward VME pairs are also more strongly coupled than the downward VME and the control pairs.

Not all gap junction connections among retinal ganglion cells (RGCs) underwent such a change. We looked at spike time correlations among OFF alpha RGCs but did not find significant difference between the control group and the VME groups (Fig 4E–4G). Thus, the effect of motion experience on gap junction connections is restricted and possibly cell type specific.

**Fig 5:**
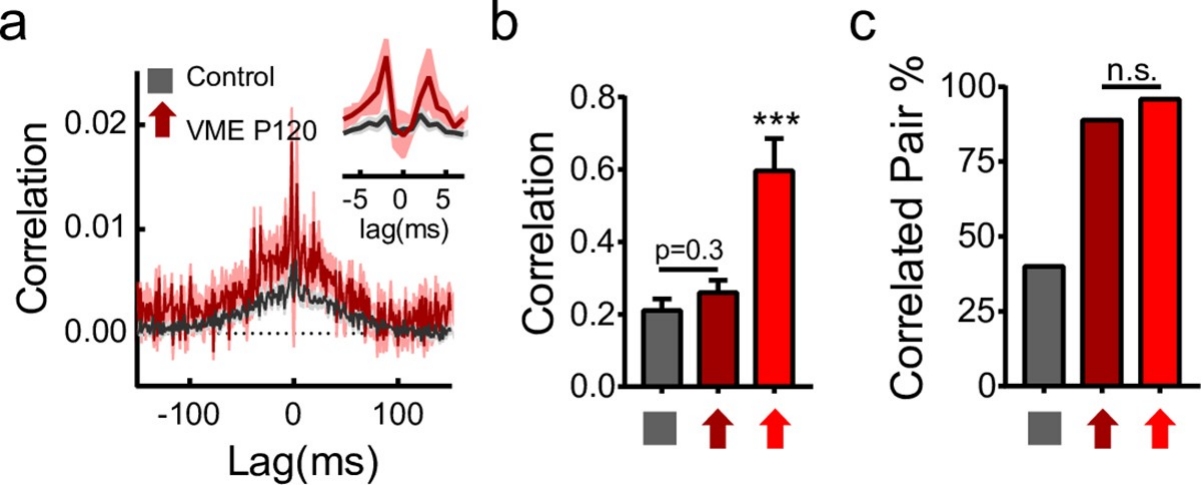
Effects of upward VME are long-term. (a) Comparison of V-DSGC cross-correlograms between control and upward gratings VME 3 months after training. Cross-correlograms of V-DSGC pairs within 250 μm of each other are averaged. Control, gray, *n* = 75; upward VME at P120, dark red, *n* = 9. Shaded areas, ±SEM. (b) Quantification of correlation for close V-DSGC pairs based on (a). Control, gray, *n* = 75; upward VME at P120, dark red, *n* = 9; upward VME, red, *n* = 24. Correlation was computed as in 2b. ***p < 0.001, unpaired *t* test. Error bars, SEM. (c) Proportion of close V-DSGC pairs that are significantly correlated. Color codes and *n* values are the same as in (b). Chi-squared test, *p* = 0.89, comparing upward VME recorded at P120, with upward VME recorded right after training. To test the effect of the smaller sample size of the P120 group, we randomly chose 9 pairs from the regular VME group and compared with the P120 group. This was repeated 1,000 times. In 100% of cases, no significant difference was detected between the two VME groups. Data for this figure are in S5 Data. n.s., not significant; V-DSGC, ventral-preferring direction selective ganglion cell; VME, visual motion experience.

We also tested how long the effect of motion experience lasted after the VME training was concluded. Mice were put back into a normal housing condition, as all the control mice, after being exposed to upward-gratings experience from P10 to P35. MEA recordings of the retinas were performed around P120. Average correlation shows a tendency to be higher than control (Fig 5A); however, it is not statistically significant (Fig 5B), possibly due to the small sample size. The proportion of significantly coupled pairs in P120 mice, however, remained as high as younger mice recorded immediately after upward VME (Fig 5C). These results suggest that the change in activity correlation among V-DSGCs induced by early VME is likely long-term and irreversible by normal visual experience later.

### Stronger gap junction connections among V-DSGCs after upward VME training

**Fig 6:**
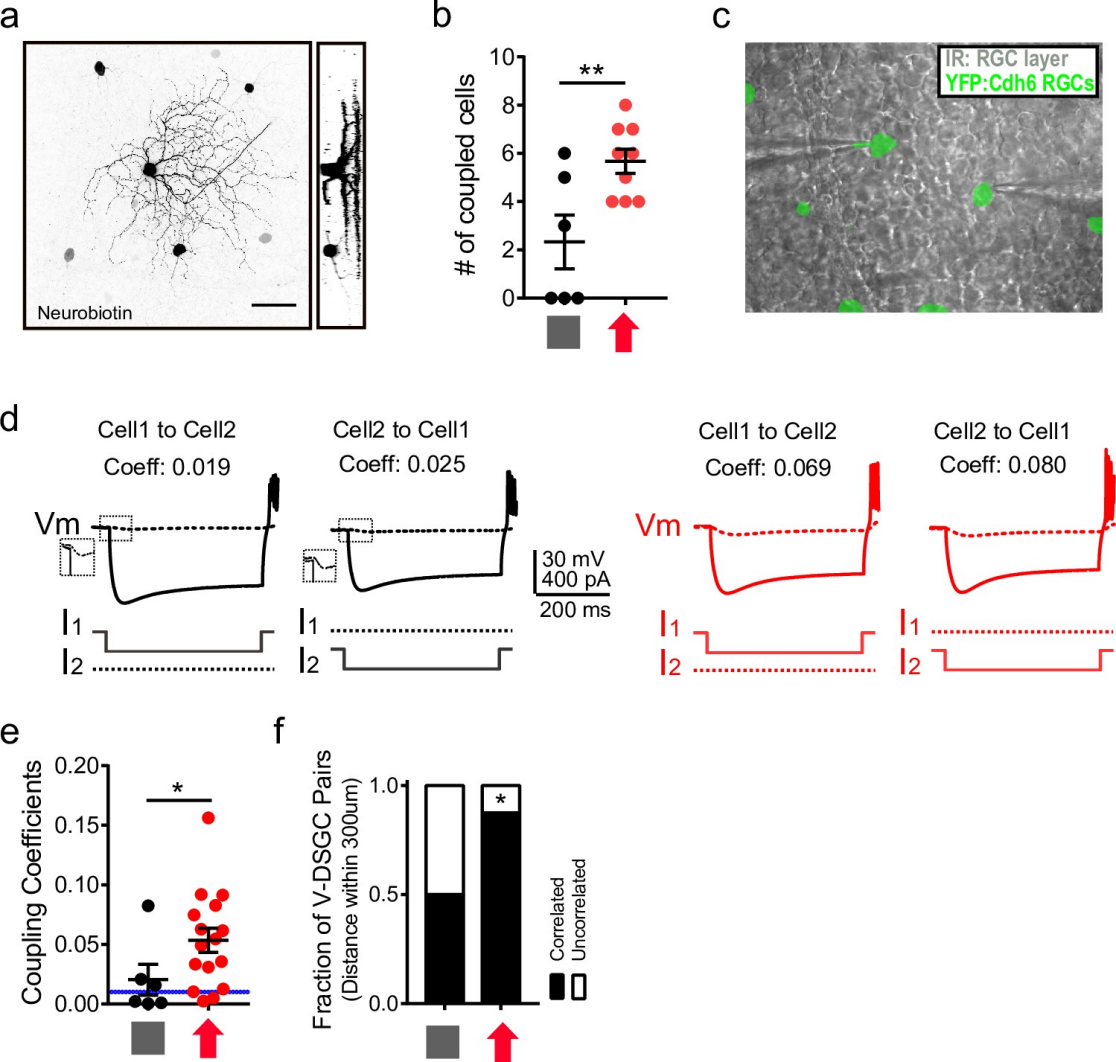
Stronger gap junction connections in V-DSGCs after upward VME. (a) Neurobiotin labeling showing dendritic arborization of a V-DSGC and the somata of neighboring RGCs that are coupled to it. Scale bar, 50 μm. (b) Number of cells coupled to V-DSGCs revealed by neurobiotin labeling of V-DSGCs. Control, 6 V-DSGCs, 2.3 ± 1.1 coupled somata; VME, 9 V-DSGCs, 5.7 ± 0.5 coupled somata. Unpaired *t* test, *p* < 0.01. (c) Face-on view of a Cdh6-CreER × Thy1-stop-YFP mouse retina captured during a paired intracellular recording session. Gray, infrared illuminated RGC layer; green, fluorescence of YFP. (d) Electrical coupling between a pair of V-DSGCs each in the control (black) and upward gratings VME (red) groups, as revealed by targeted paired intracellular recording in the Cdh6-CreER × Thy1-stop-YFP retina. In both cases, −200 pA current injection in cell 1 caused hyperpolarization in cell 2 and vice versa. Coupling coefficients are indicated on top. (e) Coupling coefficients for V-DSGC pairs. Control, 6 pairs, mean coupling coefficients 0.02 ± 0.01; VME, 16 pairs, mean coupling coefficients 0.05 ± 0.01. Dashed line shows the threshold for significant coupling. Unpaired *t* test, *p* < 0.05. (f) Fraction of electrically coupled V-DSGCs. Control, 50%, 6 pairs; VME, 87.5%, 16 pairs. Chi-squared test, *p* < 0.05. Data for this figure are in S6 Data. IR, infrared; RGC, retinal ganglion cell; V-DSGC, ventral-preferring direction selective ganglion cell; Vm, membrane potential; VME, visual motion experience; YFP, yellow fluorescent protein.

Changes in synchronized activity suggest that the gap junction connections among V-DSGCs have been modulated by VME training. We tested this directly in two ways: a tracer coupling experiment to visualize the number of V-DSGCs coupled to a single V-DSGC; and paired intracellular recordings of V-DSGCs to test the strength of the electrical coupling. Cdh6-CreER × Thy1-stop-YFP mice [30], where V-DSGCs are fluorescently labeled, were used for visual motion training and subsequent recordings. All V-DSGCs in the upward gratings VME group filled with neurobiotin showed coupling to a group of 3 to 8 RGCs (Fig 6A), while only half of the control V-DSGCs showed comparable coupling, with the other half showing no detectable tracer coupling to other RGCs (Fig 6B).

From paired current clamp recordings (Fig 6C and 6D), the mean coupling coefficient between V-DSGCs in the VME group was about twice as high as that of the control group (Fig 6E). This is mostly due to an increase in coupled pairs: about half of the V-DSGC pairs in the control group showed significant electrical coupling (coupling coefficient >0.01 [31], Fig 6F), whereas 87.5% of pairs in the VME group are electrically coupled. These results indicate that significantly more V-DSGCs are electrically coupled with each other in the upward VME retina. They are also consistent with gap junction connections between V-DSGCs being potentiated by early training with upward VME.

## Discussion

The main finding of this study is that after exposing the mice to a visual environment enriched in upward motion for a prolonged time right after eye-opening, gap junction connections among upward preferring DSGCs (V-DSGCs) are significantly potentiated, while early exposure to downward motion has the opposite effect. This outcome is tightly linked to the level of synchronized firing in V-DSGCs during the visual training. The change is long-term; it persists three months after the training stimulus has been removed. Our results demonstrate that gap junction connections in the retina are involved in experience-dependent plasticity during early development.

### Connection numbers, connection strength, and potential mechanism

The results presented in this report indicate that both the number of gap junction–connected V-DSGCs and the connection strength are enhanced by upward VME training, which then lead to an elevated level of spike synchrony between these cells (Fig 4). However, the increase in the proportion of connected cells likely accounts for a major part of the observed enhancement in spike synchrony, while the strengthened gap junction connections seem to play a slightly more minor role (Fig 4B vs 4D). Our MEA recording can record V-DSGC pairs whose soma-to-soma distances range from 50 to 500 μm. Because the strength of gap junction connections decreases with increasing distance between cells, one needs to heed the distribution of pair distance within a population of V-DSGCs when comparing average cross-correlations between different populations. To restrict the influence of the distance, we focused much of our comparison of correlation on pairs that are within 250 μm of each other, thus having a significant proportion of overlapping dendritic fields and, consequently, stronger coupling. However, even if we compare correlation for pairs that are further apart than 250 μm, both the proportion of correlated pairs and the strength of the correlation are similarly impacted by VME training. These observations suggest that V-DSGCs in the upward VME group are much more likely to connect with V-DSGCs via gap junctions, and with stronger connection strength as well. Within the limit set by our recording method, distance between cell pairs does not seem to be a limiting factor on whether VME training may influence their gap junction connections.

When inferring gap junction connection strength from spike time correlation, one should be mindful that weak connections might go undetected due to the limited number of spikes used in the correlation analysis. Taking into account this limitation, we propose that most V-DSGCs within the contact range of their dendrites form gap junction connections. In the control retina, after normal development, many of these gap junction connections are weak or inactive; only a portion of V-DSGC pairs, mostly those that are close to each other, with large overlap of dendritic fields, have enough active gap junction connections to exhibit measurable spike synchrony. When the mice are exposed to a visual environment such as those dominated by downward moving dots or vertical moving dots from eye-opening, activity synchronization through these gap junction connections is minimal or possibly suppressed (Fig 3A), while general activity level is normal or even higher (vertical dots). Through a Hebbian-like mechanism, this may lead to gradual depression of the gap junction connections, to the extent that no measurable coupling can be detected via spike synchrony post-development (Fig 2C). Conversely, with upward motion training, gap junction–mediated activity correlation is elevated in all V-DSGCs for a prolonged period, resulting in a general enhancement of gap junction connection strength among V-DSGCs. This manifests as more coupled cells, as well as stronger connections between. For pairs that are farther apart, with very small overlap of dendritic fields, and that do not show measurable connections under normal circumstances, potentiated connection may put the pair into the “connected” category with a very low measurement of total connection strength. This hypothesis considers the effects on connection numbers and connection strength being not fundamentally different from each other, that potentiated connection naturally leads to a higher percentage of pairs being considered connected. More studies such as the time course of the change in gap junction connections and the expression and localization of gap junction proteins during training are needed to test if the hypothesis is correct and to elucidate the mechanism behind this experience-dependent gap junction plasticity during development.

There are discrepancies between our gap junction coupling strength measurements (Fig 6) and those from previous studies [23,32]. Our measurements tend to be weaker: In control retina, we see on average 2–3 tracer coupled cells, and coupling strength varies from 0 to 0.08, with a mean value at approximately 0.02, whereas Trenholm and colleagues reported 5–7 coupled cells on average, and the coupling strength can be as high as 0.2. Potential explanations for these discrepancies include the adaptational state of the retina [12] and the distance between recorded pairs. We dark-adapted the animal before experiments to preserve the light response, while Trenholm and colleagues light-adapted the animal to enhance gap junction coupling. This and differences in tracer coupling protocols may account for the discrepancy in our tracer coupling results. In addition, different transgenic animals were used: The distances between our recorded V-DSGC pairs vary from 70 to 250 μm using Cdh6-CreER × Thy1-stop-YFP animals, while pair distances in the Hb9::eGFP retina Trenholm and colleagues recorded from appear to be more uniform, and much smaller, thus potentially have systematically stronger coupling. In our experiments, we recorded from both control and VME retinas under exactly the same condition, and oftentimes on the same day using the same reagents. Thus, despite these discrepancies in absolute values of correlation strength, we believe the direction of change we observed between VME and control groups is consistent and valid.

On closer examination of the response properties of V-DSGCs after VME training, it appears that first-order statistics such as absolute firing rates and autocorrelation vary between different VME groups (S1 Table and S5 Fig). In part, this may simply reflect variations in recording quality and noise. Nevertheless, there is a tendency for groups with higher cross-correlation to also have higher firing rates. We normalized the cross-correlation to the product of the spike numbers, so that the correlation value is independent of absolute activity levels. VME-induced changes in cross-correlation were still observed. Thus, the change in correlation is not caused by the change in firing rates. The question becomes whether the reverse is true, that enhanced coupling leads to elevated activity levels. Due to the nature of the electrical coupling, a network of neurons with enhanced homologous coupling among them has the potential to show increased activity level. More detailed study using gap junction blocker or gap junction protein knockout animals is needed to definitively answer this question.

### Why V-DSGCs, upward motion, and not others

In the visual cortex, experience-dependent plasticity takes on a slightly different form. Motion training using any direction of motion can induce rapid emergence of DS columns for the trained direction [33]. Similarly, early experience with any restricted orientation results in overrepresentation of that orientation in V1 [34]. There does not appear to be a bias for neurons preferring certain directions or orientations to be affected more strongly than the others, whereas from the results in this report, only gap junction connections between V-DSGCs are affected. For gap junctions in the other subtypes of ooDSGCs, they are still present at the beginning of VME training, yet they disappear after eye-opening, with or without VME training. Thus, developmentally regulated expression of gap junctions in these other ooDSGCs are not impacted; only gap junction connection strength among V-DSGCs is affected by VME. It is worth noting, however, that cortical neurons differ in their sensitivity to early visual experience too: In ferret V1, DS can be rapidly induced by motion experience, but orientation selectivity (OS) only shows much weaker enhancement [33]. In the mouse visual cortex, OS is plastic to changes induced by altered visual experience, whereas DS is already mature at eye-opening [34,35]. One obvious explanation for such differences is that the developmental processes of the corresponding neural circuits are not completely synchronized, and visual experience may have stronger impact on those circuits that develop at a slower pace and thus more plastic during later stages of development.

Our results demonstrated a tight correlation between the level of correlated activity during training and the change in gap junction connection strength. The stronger the correlation during training, the more potentiation. Conversely, if the training decorrelates the V-DSGCs, then the gap junction connections are weakened. This strongly suggests that correlated activity in the connected V-DSGCs directly leads to changes in the gap junction connection. Direct examination of gap junction connections in V-DSGCs around eye-opening should provide more insights into this type of plasticity. Even if we answer the “how” question, the “why” question remains: Why is V-DSGC the only subtype of ooDSGCs that keep gap junction connections among them? These connections can even be modulated by visual experience during early development, implying a level of functional importance. Is there a functional or ethological reason that mice treat upward motion–preferring ooDSGCs, or upward motion in visual inputs, differently than the others? This is a question that also warrants attention.

### Potential implications

Gap junction connections among neurons play critical roles during development. These connections are prominent before P12 in the mouse brain but decline afterward with the emergence of chemical synapses [36]. It is proposed that the early gap junction connections among neurons might help to establish a developmental blueprint, affecting the formation of the mature neural circuits [37]. Our work presented here is an example of how electrical synapses, through regulation of synchronized firing between neurons, may influence the neural circuits that harbor these synapses, potentially also exert impacts on the chemical synapses within the circuits, and consequently modulate the circuit functions.

Electrical synapses exhibit extensive plasticity, and the vertebrate retina is a popular place to study it [38,39]. In this work, we present another example of gap junction plasticity. Activity through the gap junction connections among ooDSGCs play an important role in inducing changes in the ooDSGC circuit. At the same time, these gap junction connections themselves are also potentiated or depressed by the process. These changes are stable and long-term. We also tested whether VME-induced change occurred in mature animals, and the answer is no. For nursing mothers that have been put into the same training environment, no change in their ooDSGCs has been found (S6 Fig). Our results implicate the involvement of gap junction in both the cause and the effect of experience-dependent plasticity in the retina; this may provide a new angle to study neural plasticity during the development of the visual system.

## Materials and methods

### Ethics statement

All experimental procedures were approved by the Institutional Animal Care and Use Committee of the Institute of Neuroscience, Chinese Academy of Sciences. IACUC approval number NA-012-2019.

### Mice

C57BL/6 mice of both sexes and mixed litters were used in each experiment. Sample sizes (number of retinas and/or cells) are indicated in the figures or figure captions.

### Visual motion training

The homemade “visual experience” cages are 50 × 25 × 40 cm in size and light-proof. One wall of the cage (50 × 40 cm) was covered by a computer monitor to provide the VME for 12 hours each day from P10 to P35. For moving dots stimuli, 300 square dots with random combinations of intensities (0–1), sizes (0.3–128°), and speeds (29–310 mm/s) travel across the screen either in the same direction or in two opposite directions (Fig 2A). Every 6 minutes, a different set of 300 dots replaces the previous set, but the direction of motion remains the same. For moving gratings stimuli, square wave gratings with random combinations of contrasts (40% to 100%), spatial periods (1.4–157° bar width), and speeds (12–120 mm/s) travel across the screen in the same direction. Every 5 to 30 seconds, a different gratings pattern replaces the previous pattern, but the direction of motion remains the same. The average intensity of the monitor was equivalent to the following photon flux values for the 3 mouse photoreceptors, each expressed at the wavelength of peak sensitivity: rod, 4.75 × 10^4^ photons/second/μm^2^ at 500 nm; M cone, 5.56 × 10^4^ photons/second/μm^2^ at 511 nm; and S cone, 1.05 × 10^3^ photons/second/μm^2^ at 370 nm.

### Tissue preparation and MEA recording

Mice were dark-adapted for at least 2 hours, then anesthetized by isoflurane and euthanized using cervical dislocation. Retina dissections were performed under infrared or dim red illumination at room temperature. The orientation of the retina was determined using landmarks of vessels on the choroid membrane [40]. Isolated retinas were incubated with Ringer’s solution (110 mM NaCl, 1 mM CaCl_2_, 2.5 mM KCl, 1.6 mM MgCl_2_, 22 mM NaHCO_3_, 10 mM D-Glucose, aerated with 95% O_2_ and 5% CO_2_. All reagents were purchased from Sigma-Aldrich, St. Louis, MO) in a light-tight container. To preserve orientation, the retina was bisected along the temporal-nasal axis. Left and right eyes and dorsal and ventral halves of the retina were always kept track of by incubating these pieces in different compartments. For recording, each retina half was cut into elongated quadrilaterals (approximately 1 by 1.5 mm) with one corner cut to unambiguously mark its orientation, then put on to the MEA (256MEA30/8iR-ITO, MCS, Reutlingen, Germany) with the ganglion cell side down. In addition, the thickness of the retina piece (much thinner on the periphery) also assisted to correctly orient it on the MEA. The retina piece was then adapted in the dark for at least 20 minutes until a steady state of spontaneous activity was achieved. Throughout the recording, the retina piece was kept between 28 and 32°C, with a perfusion speed of 4–6 mL Ringer’s solution/minute. Extracellular spikes were recorded by the USB-MEA256-System (MCS) at a sampling rate of 10 kHz. Spike sorting was done offline by a customized program (Courtesy of Ofer Mazor, with modifications). All other analysis was performed in Matlab.

### Visual stimulation

Visual stimuli were delivered from a computer-driven LED computer monitor to the photoreceptor layer of the retina piece through a custom-made lens system (frame rate, 60 Hz; magnification, 8 μm/pixel). White light was used, and the average intensity for all stimuli was equivalent to the following photon flux values for the 3 mouse photoreceptors, each expressed at the wavelength of peak sensitivity: rod, 3.16 × 10^3^ photons/second/μm^2^ at 500 nm; M cone, 3.71 × 10^3^ photons/second/μm^2^ at 511 nm; and S cone, 6.99 × 10^1^ photons/second/μm^2^ at 370 nm. The speed series of motion stimuli are travelling gratings with a 19.2° spatial period and 100% grating contrast. The contrast series of motion stimuli are travelling gratings with a 19.2° spatial period and 24°/second motion speed.

### Identification of ooDSGCs

DS of all RGCs was measured using a full field square-wave gratings stimulus moving in eight directions (spatial frequency, 19.2°/cycle; speed 12, 24, 48°/second). An RGC was classified to be an ooDSGC if it is an ON-OFF cell, its preferred directions under all 3 speeds are within a 60° range, and its vector sum at 24°/seconds exceeds 0.1 and DSI > 0.3. Vector sum and DSIs were computed as described previously [41]. About 6–20 ooDSGCs were identified from each piece of retina. Preferred directions of ooDSGCs from a single piece of retina always cluster nicely in the four cardinal directions. DSGCs are transient RGCs; their response to a flash stimulus generally lasts less than 300 ms after the onset of the flashes. Their activity from 1 to 2 seconds after the flash onset is considered to not be affected by the stimulus and thus be the basal activity of the DSGCs. Distance between V/V pairs was deduced from the distance between electrodes on which the corresponding V-DSGCs were recorded. Because the dendritic field diameter of V-DSGCs generally does not exceed 250 μm, pair distances below 250 μm were considered “close pairs.”

### Histology and microscopy

Retinas with neurobiotin (Vector Laboratories, Burlingame, CA) filled V-DSGC(s) were fixed in 4% PFA (Sinopharm Chemical Regent, Shanghai, China) for 1 hour at room temperature, then incubated with streptavidin (1:1,000, DyLight 549 Streptavidin, Cat. No: SA-5549, Vector Laboratories, Burlingame, CA) overnight at 4°C. Confocal microscopy was then performed to visualize the tracer coupling patterns.

### Cross-correlation

Cross-correlation was computed as previously reported [42]. Each “trial” lasts 2.8 seconds, which is the time it takes for a white dot to move across the MEA area once, in a single direction of motion. A total of 80 trials (8 directions of motion, 10 repeats each) were presented to each retina piece. The geometric mean of the pair’s spike numbers was used to normalize the result. The time bin was 1 ms for all except in Fig 3A (bin size, 25 ms), S2 Fig (bin size, 10 ms), and S3 Fig (bin size, 25 ms). In all cases except those specifically noted in the text, the shift predictor (cross-correlation using shuffled repeats) [42] was subtracted from the raw cross-correlation, removing the part that is induced by inherent correlation within the stimulus structure. The remaining correlation is generally considered to be the product of the circuit function, such as gap junction connections. In S2D Fig and S3 Fig, correlation was normalized to the product of spike numbers. This normalization method combined with shift predictor subtraction produces correlation values that are invariant to changes in the average firing rates of the cells [27]. If the peak of a cross-correlation deviated by at least three standard deviations from the baseline (time lag between −500–−100 ms and 100–500 ms), the pair was considered to have significant correlation. Total correlation value was calculated as the sum of the cross-correlation of the time lag from −100 ms to 100 ms (−15 ms to 15 ms for OFF αRGCs).

### Intracellular recording

Tissue preparation was the same as that for MEA recording. For functional identification of ooDSGCs, cell-attached recordings were performed using 5–8 MΩ electrodes (World Precision Instruments, Sarasota, FL) filled with Ringer’s solution. To study gap junctions between V-DSGCs, paired current clamp recording was performed. Internal solution was as follows (in mM): 120 potassium gluconate, 5 NaCl, 10 KCl, 1 MgCl_2_, 1 EGTA, 10 HEPES, 2 ATP-Na_2_, 0.5 GTP-Na_3_, and 10 neurobiotin (cell filling); the pH was adjusted to 7.2 with KOH; and resistance of electrodes was 4–6 MΩ. All reagents for the internal solution (except neurobiotin) were purchased from Sigma-Aldrich, St. Louis, MO. Current injection, at 0.67 Hz, with 500 ms pulse duration and −200 pA pulse amplitude, was repeated 10 times in each cell to quantify the strength of gap junction connections. Data were acquired by a MultiClamp 700B amplifier (Molecular Devices, San Jose, CA), low-pass filtered at 2 kHz and digitized at 10 kHz. Gap junction coupling coefficients were calculated as described previously [43]. According to previous reports [31,44], neuronal pairs with coupling coefficient >0.01 are considered to be coupled. Neurobiotin injection was performed under the current clamp after functional identification by cell-attached recordings. Positive current injection (100 pA, 2 Hz, 15 minutes) followed by 30 minutes of incubation was performed to facilitate neurobiotin diffusion before the retina was taken for staining.

### Statistical analysis

Specific methods used are indicated in the main text or figure captions.

## Supporting information

S1 Fig Electrical coupling among ooDSGCs in adult retina. (a-c) Spike time cross-correlograms for D/D, T/T, and N/N ooDSGC pairs in adult mice (P42–P56). Solid black line, average cross-correlogram across all pairs of the same combination. Shaded area, SEM. For each panel, the inset on the left indicates the ooDSGCs pair; the inset on the right is a close-up view of the cross-correlogram with a 1-ms bin size. D-D, *n* = 222; N-N, *n* = 8; T-T, *n* = 172. Data for this figure are in S1 Data. D, dorsal-preferring direction selective ganglion cell; N, nasal-preferring direction selective ganglion cell; ooDSGC, ON-OFF direction selective ganglion cell; T, temporal-preferring direction selective ganglion cell; V, ventral-preferring direction selective ganglion cell
(TIF)

S2 Fig Spike time cross-correlation for spontaneous activity. (a) Comparison of spontaneous activity levels in the upward VME, downward VME, and control groups. (b) Average cross-correlograms from shuffled trials (shift predictors) of stimulus-evoked (blue) and spontaneous (black) activity from the upward VME group. The effect of the stimulus (a white dot moving across the MEA in 8 directions) is apparent in its shift predictor. As a comparison, the shift predictor for the spontaneous activity (arbitrary 1-second “trials”) is a structureless flat line. (c) Mean cross-correlograms from spontaneous spikes for the upward VME, downward VME, and control groups. The results are qualitatively the same as stimulus-evoked correlation in the 3 groups; in other words, upward dots VME > control > downward dots VME. (d) The same as (c) but normalized to the product of spike numbers to remove the effect of firing rate on the measurement of cross-correlation. Even accounting for the 2-fold change in basal activity levels between upward VME and control shown in (a), there is still significant difference in cross-correlation between the 3 groups. The trend remains the same as in (c). *n* = 50/42/48 V-DSGCs for control/upward VME/downward VME groups in (a), and *n* = 139/51/130 pairs for control/upward VME/downward VME groups, respectively, in (b), (c), and (d). Data for this figure are in S2 Data. MEA, multielectrode array; V-DSGC, ventral-preferring direction selective ganglion cell; VME, visual motion experience.
(TIF)

S3 Fig Stronger correlation among V-DSGCs during upward motion. Polar plot showing levels of correlated activity induced by different directions of motion stimuli. Correlation was normalized to the product of firing rates to minimize the effect of activity level on correlation. Upward motion induces much higher spike time correlation than the downward directions. Data for this figure are in S3 Data. V-DSGC, ventral-preferring direction selective ganglion cell.
(TIF)

S4 Fig Absolute activity level induced by the VME training stimulus is not directly connected with the effect of VME. (a) Response of normal (untrained) V-DSGCs in response to the VME training stimuli, downward gratings, and vertical dots. *N* = 24 V-DSGCs, paired *t* test, *p* < 0.001. V-DSGCs respond more strongly to vertical dots than to downward gratings. (b) Correlation between V-DSGC pairs after being trained by vertical dots VME or downward gratings VME. Training with vertical dots VME resulted in weaker correlation compared to training with downward gratings VME. Numbers of V-V pairs: 253 for vertical dots VME group, 55 for downward gratings VME group. Unpaired *t* test, *p* < 0.001. Data for this figure are in S3 Data. V-DSGC, ventral-preferring direction selective ganglion cell; VME, visual motion experience.
(TIF)

S5 Fig Lower-order statistics of V-DSGCs from different VME groups. The dataset is the same as that used in Fig 3E–3H. The basal firing rates (a), peak firing rates in response to the moving dot stimulus (b), and the average autocorrelations (c) are shown. The autocorrelation is symmetric around 0, so only one side is shown. Data for this figure are in S3 Data. V-DSGC, ventral-preferring direction selective ganglion cell; VME, visual motion experience.
(TIF)

S6 Fig VME training has no effect in adult animals. (a, b) Comparison of V-DSGC cross-correlation between the control and adult training (upward VME) groups. Adult mice were exposed to the same upward VME training as the developing pups for the same amount of time. Both average cross-correlograms (a) and correlations (b) are compared. Correlation was computed as in Fig 2B. Control, gray, *n* = 139 pairs of V-DSGCs; upward VME (Adult), dark magenta, *n* = 15. Shaded areas in (a), ±SEM. Error bars in (b), SEM. Unpaired *t* test, *p* = 0.52. (c) Comparison of the proportions of V-DSGC pairs that show significant spike time correlation. Color codes and *n* values are the same as in (a). Chi-squared test, *p* = 0.37. Data for this figure are in S5 Data. V-DSGC, ventral-preferring direction selective ganglion cell; VME, visual motion experience.
(TIF)

S1 Table Summary on the datasets used in cross-correlation studies. (PDF)

S1 Data Supplementary excel file for data presented in Fig 1 and S1 Fig. The file has multiple sheets, labeled by figures and panels.
(XLSX)

S2 Data Supplementary excel file for data presented in Fig 2 and S2 Fig. The file has multiple sheets, labeled by figures and panels.
(XLSX)

S3 Data Supplementary excel file for data presented in Fig 3 and S3–S5 Figs. The file has multiple sheets, labeled by figures and panels.
(XLSX)

S4 Data Supplementary excel file for data presented in Fig 4. The file has multiple sheets, labeled by panels.
(XLSX)

S5 Data Supplementary excel file for data presented in Fig 5 and S6 Fig. The file has multiple sheets, labeled by figures and panels.
(XLSX)

S6 Data Supplementary excel file for data presented in Fig 6. The file has multiple sheets, labeled by panels.
(XLSX)

## Abbreviations

D-DSGC: dorsal-preferring direction selective ganglion cell
DS: direction selectivity
DSGC: direction selective ganglion cell
MEA: multielectrode array
N-DSGC: nasal-preferring direction selective ganglion cell
ooDSGC: ON-OFF direction selective ganglion cell
OS: orientation selectivity
P: postnatal day
RGC: retinal ganglion cell
T-DSGC: temporal-preferring direction selective ganglion cell
V-DSGC: ventral-preferring direction selective ganglion cell
VME: visual motion experience
18β-GA: 18β-glycyrrhizic acid
