# Supplementary figures and images for: Early visual motion experience shapes the gap junction connections among direction selective ganglion cells
Source: PLoS Biol. 2020 Mar 25;18(3):e3000692. doi: 10.1371/journal.pbio.3000692 (PMC7135332; doi:10.1371/journal.pbio.3000692)

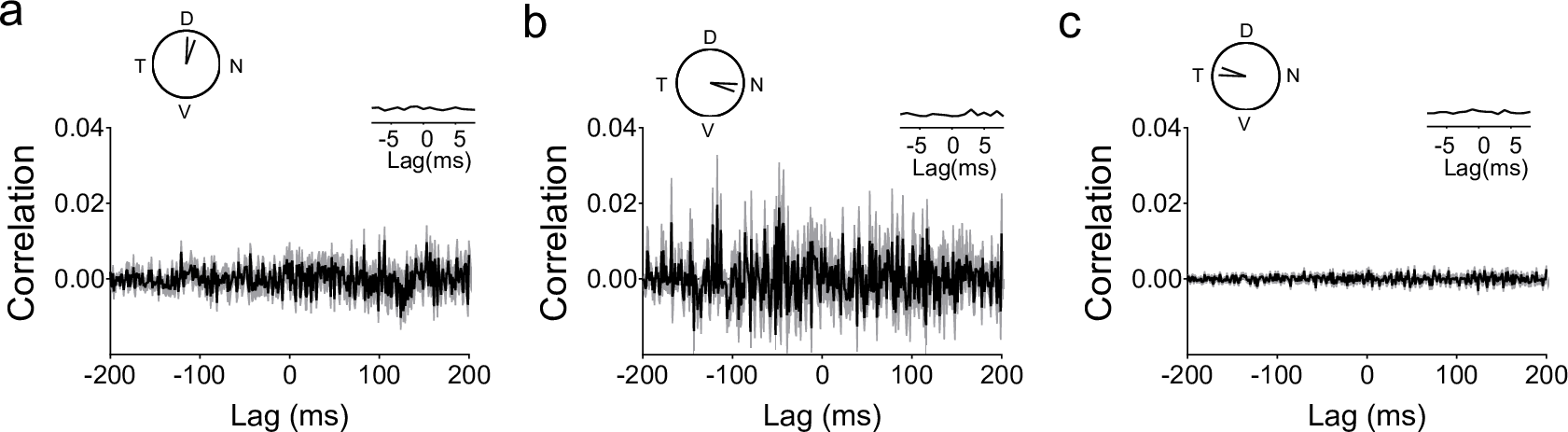

Supplement: S1 Fig — (a-c) Spike time cross-correlograms for D/D, T/T, and N/N ooDSGC pairs in adult mice (P42–P56). Solid black line, average cross-correlogram across all pairs of the same combination. Shaded area, SEM. For each panel, the inset on the left indicates the ooDSGCs pair; the inset on the right is a close-up view of the cross-correlogram with a 1-ms bin size. D-D, n = 222; N-N, n = 8; T-T, n = 172. Data for this figure are in S1 Data. D, dorsal-preferring direction selective ganglion cell; N, nasal-preferring direction selective ganglion cell; ooDSGC, ON-OFF direction selective ganglion cell; T, temporal-preferring direction selective ganglion cell; V, ventral-preferring direction selective ganglion cell (TIF) [file pbio.3000692.s001.tif]

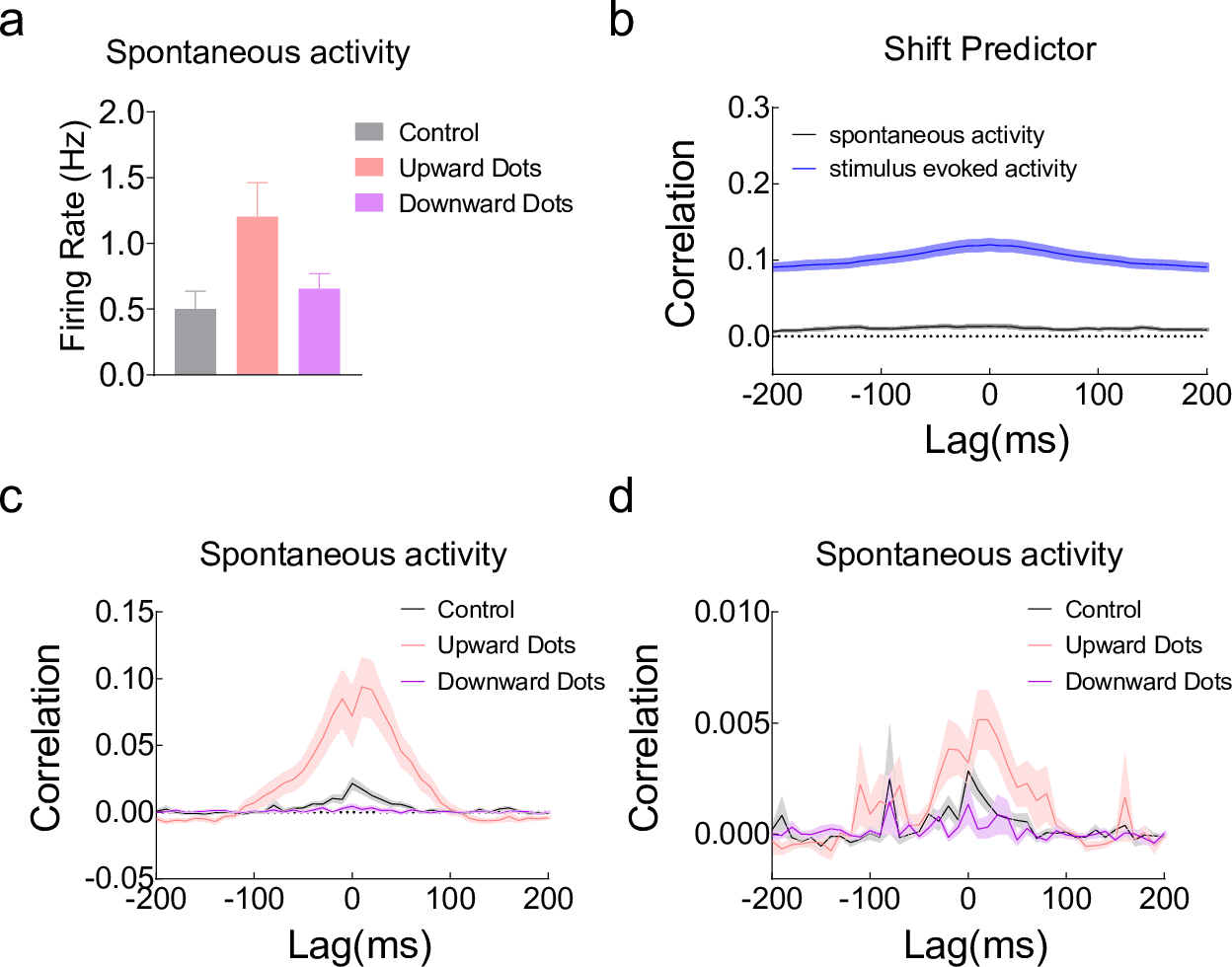

Supplement: S2 Fig — (a) Comparison of spontaneous activity levels in the upward VME, downward VME, and control groups. (b) Average cross-correlograms from shuffled trials (shift predictors) of stimulus-evoked (blue) and spontaneous (black) activity from the upward VME group. The effect of the stimulus (a white dot moving across the MEA in 8 directions) is apparent in its shift predictor. As a comparison, the shift predictor for the spontaneous activity (arbitrary 1-second “trials”) is a structureless flat line. (c) Mean cross-correlograms from spontaneous spikes for the upward VME, downward VME, and control groups. The results are qualitatively the same as stimulus-evoked correlation in the 3 groups; in other words, upward dots VME > control > downward dots VME. (d) The same as (c) but normalized to the product of spike numbers to remove the effect of firing rate on the measurement of cross-correlation. Even accounting for the 2-fold change in basal activity levels between upward VME and control shown in (a), there is still significant difference in cross-correlation between the 3 groups. The trend remains the same as in (c). n = 50/42/48 V-DSGCs for control/upward VME/downward VME groups in (a), and n = 139/51/130 pairs for control/upward VME/downward VME groups, respectively, in (b), (c), and (d). Data for this figure are in S2 Data. MEA, multielectrode array; V-DSGC, ventral-preferring direction selective ganglion cell; VME, visual motion experience. (TIF) [file pbio.3000692.s002.tif]

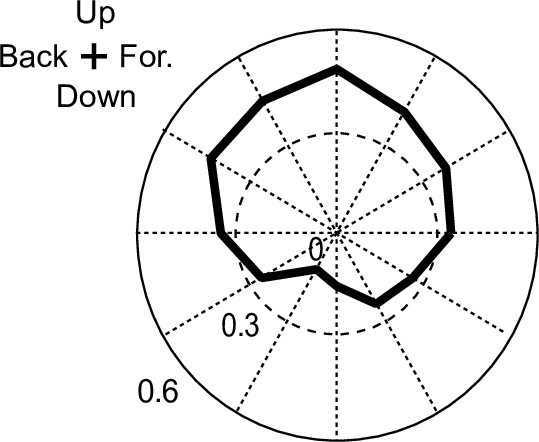

Supplement: S3 Fig — Polar plot showing levels of correlated activity induced by different directions of motion stimuli. Correlation was normalized to the product of firing rates to minimize the effect of activity level on correlation. Upward motion induces much higher spike time correlation than the downward directions. Data for this figure are in S3 Data. V-DSGC, ventral-preferring direction selective ganglion cell. (TIF) [file pbio.3000692.s003.tif]

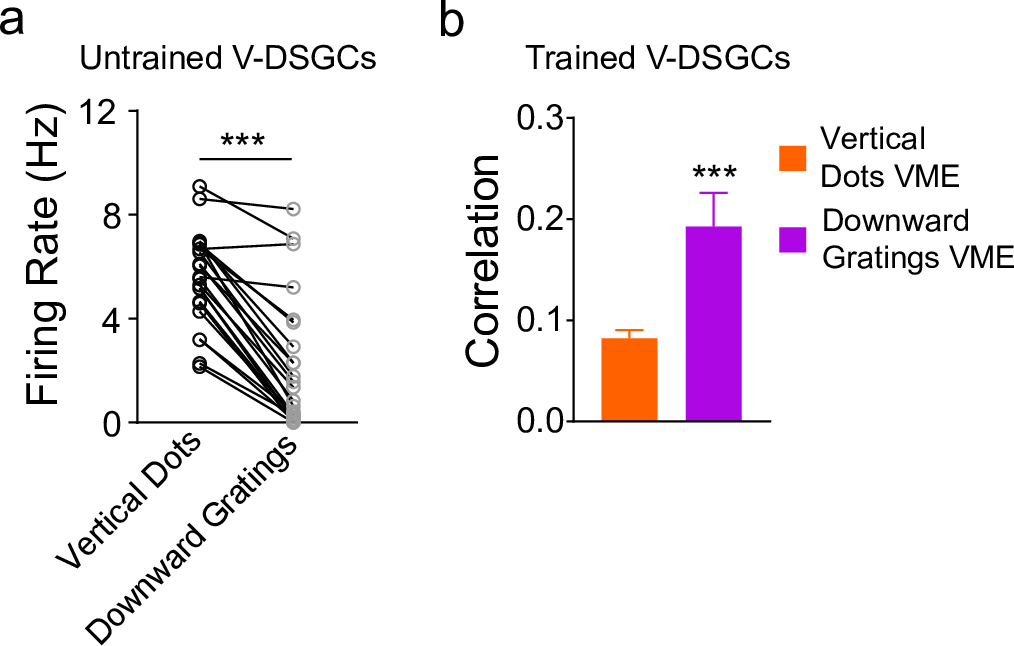

Supplement: S4 Fig — (a) Response of normal (untrained) V-DSGCs in response to the VME training stimuli, downward gratings, and vertical dots. N = 24 V-DSGCs, paired t test, p < 0.001. V-DSGCs respond more strongly to vertical dots than to downward gratings. (b) Correlation between V-DSGC pairs after being trained by vertical dots VME or downward gratings VME. Training with vertical dots VME resulted in weaker correlation compared to training with downward gratings VME. Numbers of V-V pairs: 253 for vertical dots VME group, 55 for downward gratings VME group. Unpaired t test, p < 0.001. Data for this figure are in S3 Data. V-DSGC, ventral-preferring direction selective ganglion cell; VME, visual motion experience. (TIF) [file pbio.3000692.s004.tif]

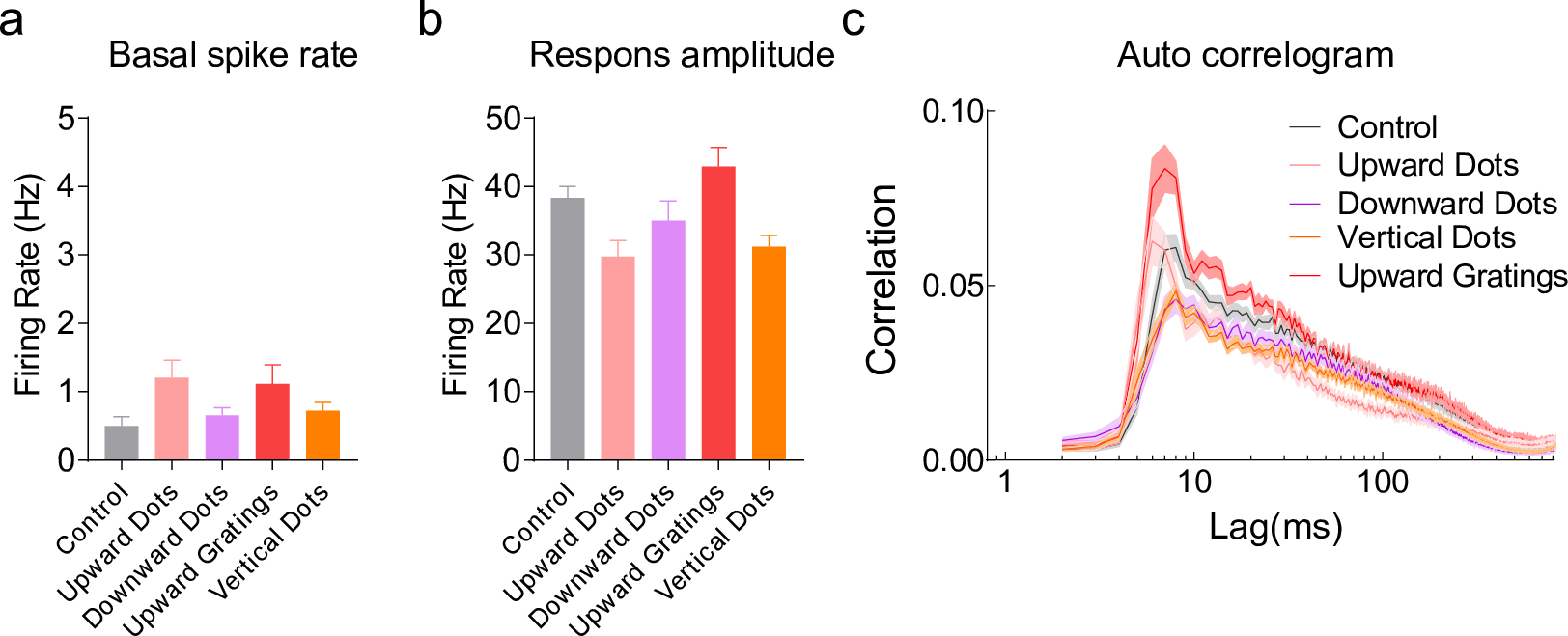

Supplement: S5 Fig — The dataset is the same as that used in Fig 3E–3H. The basal firing rates (a), peak firing rates in response to the moving dot stimulus (b), and the average autocorrelations (c) are shown. The autocorrelation is symmetric around 0, so only one side is shown. Data for this figure are in S3 Data. V-DSGC, ventral-preferring direction selective ganglion cell; VME, visual motion experience. (TIF) [file pbio.3000692.s005.tif]

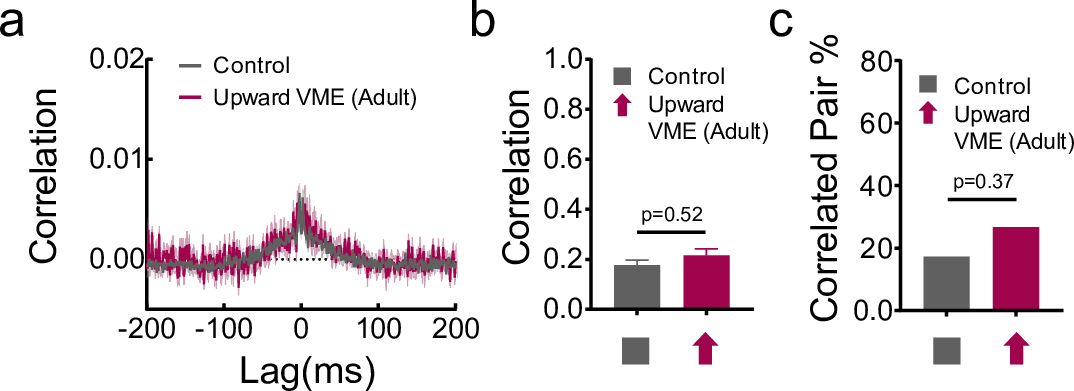

Supplement: S6 Fig — (a, b) Comparison of V-DSGC cross-correlation between the control and adult training (upward VME) groups. Adult mice were exposed to the same upward VME training as the developing pups for the same amount of time. Both average cross-correlograms (a) and correlations (b) are compared. Correlation was computed as in Fig 2B. Control, gray, n = 139 pairs of V-DSGCs; upward VME (Adult), dark magenta, n = 15. Shaded areas in (a), ±SEM. Error bars in (b), SEM. Unpaired t test, p = 0.52. (c) Comparison of the proportions of V-DSGC pairs that show significant spike time correlation. Color codes and n values are the same as in (a). Chi-squared test, p = 0.37. Data for this figure are in S5 Data. V-DSGC, ventral-preferring direction selective ganglion cell; VME, visual motion experience. (TIF) [file pbio.3000692.s006.tif]
