# Supplementary material for: Early visual motion experience shapes the gap junction connections among direction selective ganglion cells
Source: PLoS Biol. 2020 Mar 25;18(3):e3000692. doi: 10.1371/journal.pbio.3000692 (PMC7135332; doi:10.1371/journal.pbio.3000692)
Supplement: S1 Table — (PDF) [file pbio.3000692.s007.pdf]

Supplementary Table 1:Summary on the datasets used in cross-correlation studies.

VME training patterns	Stimulus used for recording	Sub-groups	n (v-DSGCs)	n(pairs)	FR±SEM	Figure panel(s)
**No training**	Upward dots	all	11	16	8.39±0.69	Fig3c,d;
	Upward gratings				8.66±0.86	
	Vertical dots				5.81±0.44	
**No training**	Moving Dots (8 directions)	all	50	139	5.23±0.33	Fig1b; Fig2b,c; Fig3e,f; FigS6a-c;
		distance<250 µm	50	75	5.23±0.33	Fig4a,b; Fig5a-c;
		distance<250 µm & correlated	23	30	6.39±0.44	Fig4c,d;
**Upward dots**	Moving Dots (8 directions)	all	42	51	5.51±0.57	Fig2b; Fig3g,h;
**Downward dots**	Moving Dots (8 directions)	all	48	130	4.19±0.41	Fig2c; Fig3g,h;
**Vertical dots**	Moving Dots (8 directions)	all	88	251	4.49±0.31	Fig3f,g,h;
**Upward gratings**	Moving Dots (8 directions)	all	16	33	6.89±0.51	Fig3e,g,h
		distance<250 µm	12	24	7.69±0.47	Fig4a,b; Fig5b,c;
		distance<250 µm & correlated	12	23	7.69±0.47	Fig4c,d;
**Downward gratings**	Moving Dots (8 directions)	distance<250 µm	13	38	6.01±0.74	Fig4a,b;
		distance<250 µm & correlated	10	11	6.33±0.59	Fig4c,d;
**Upward gratings #**	Moving Dots (8 directions)	distance<250 µm	5	9	6.80±0.53	Fig5a-c;
**Upward gratings ##**	Moving Dots (8 directions)	all	6	15	4.64±0.57	FigS6a-c;

V-DSGCs: Ventral preferring DSGC; FR: firing rate(Hz).
All VME groups were trained from p10 to p35, recorded at p35-p56, except # and ##:
Training: P10-P35, recording: P120; ## Training at adult age, recording after training.
Specific methods for the selection of correlated pairs and close (distance<250µm) pairs are described in the method section.
